# Evaluating the Effectiveness of Insulin Plus Oral Medications Versus Oral Anti‐Diabetes Therapy Alone in Patients With Newly Diagnosed Type 2 Diabetes With Very High hbA1c and Acute Coronary Syndrome

**DOI:** 10.1002/edm2.70069

**Published:** 2025-06-07

**Authors:** Adeel Ahmad Khan, Fateen Ata, Afia Aziz, Aya Janan Qassim, Ahmed Shukri, Khaled Abdallah Aboujabal, Yazan Almohtasib, Amin Jayyousi, Mohammed Bashir, Haval Surchi

**Affiliations:** ^1^ Department of Internal Medicine Cleveland Clinic Akron General Akron Ohio USA; ^2^ Department of Endocrinology Hamad Medical Corporation Doha Qatar; ^3^ Department of Internal Medicine Hamad Medical Corporation Doha Qatar; ^4^ Department of Internal Medicine Texas Tech University Health Science Center Lubbock Texas USA; ^5^ Department of Internal Medicine University of Missouri – Kansas City Kansas City Missouri USA

**Keywords:** acute coronary syndrome, newly diagnosed type 2 diabetes mellitus, uncontrolled hyperglycaemia

## Abstract

**Introduction:**

Many patients with Acute Coronary Syndrome (ACS) are newly diagnosed with Type 2 Diabetes Mellitus (T2DM) with very high hbA1c levels (> 10%). Early achievement of glycaemic control is of prime importance in such cases, and many guidelines recommend starting insulin together with oral anti‐diabetic drugs (OAD) as part of discharge medications. However, large numbers of treatment‐naïve patients are hesitant to use insulin due to various factors.

**Methods:**

In this retrospective, single‐centre, observational study, we compared the hbA1c at 1‐year follow‐up between newly diagnosed DM patients with initial hbA1c > 10% who were discharged on insulin plus OAD versus those only on OAD after admission with ACS. Pairwise comparisons between continuous and categorical study variables were performed using t‐test, Mann–Whitney test, and chi‐square. We used STATA 18 for analysis. Baseline characteristics have been described for all the patients included in the study. In the analysis of outcomes at follow‐up, only patients who had follow‐up at 1‐year were included.

**Results:**

Of 149 patients eligible for inclusion, the majority were males (97.3%). The mean age was 47 ± 8.3 years. The baseline hbA1c at diagnosis was 11.2 (10.5–12.3) %. 38 (25.5%) Were Discharged on insulin + OADs, whereas 111 (75.5%) Were Discharged Only on OADs. There was no statistically significant difference in change in hbA1c from baseline between the two groups (Mean (SD) 4.4% ± 1.8% vs. 4% ± 1.5%, *p* = 0.07). None of the patients had any hyperglycaemic emergency, and there were no differences in recurrent admissions due to cardiac indications (*p* = 0.5).

**Conclusion:**

An anti‐DM regimen consisting of multiple oral agents is a safe and effective alternative to insulin plus OAD and can lead to a comparable reduction in hbA1c at 1‐year in patients who are not willing to use insulin early after diagnosis of T2DM.

## Graphical abstract

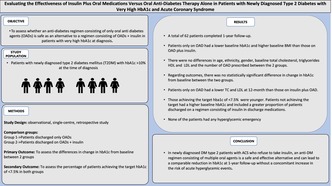

## 1 Introduction

Type 2 diabetes mellitus (T2DM) is one of the leading chronic diseases in the world, affecting around 422 million people worldwide and being directly responsible for up to 1.5 million deaths per year [1]. Atherosclerotic cardiovascular disease (ASCVD) is the most common cause of morbidity and reduced life expectancy in patients with T2DM, affecting 32.2% of patients [2]. The relative risk of myocardial infarction (MI) is 50% higher in men with diabetes and 150% higher in women with diabetes. Furthermore, there is an increased risk of recurrence of MI (40%) in patients with T2DM [3].

A significant number of patients remain undiagnosed with T2DM and are diagnosed only at the time of hospital encounter for some other indication. Ding et al. reported the prevalence of newly diagnosed DM to be 14.3% in patients diagnosed with acute MI [4]. Another study by Umar et al. noted that 29.6% of patients admitted with acute MI were newly diagnosed with DM [5]. Management of T2DM involves a stepwise approach, starting with initiating oral antihyperglycemic drugs, especially metformin, as the first‐line option. Adding insulin therapy is considered as an augmentation therapy if glycaemic targets are not met. However, in certain situations, the addition of insulin as a first‐line antihyperglycemic treatment should be considered. In the American Association of Clinical Endocrinologists (AACE)/American College of Endocrinology (ACE) comprehensive T2DM 2017 management algorithm, insulin is recommended for T2DM patients presenting with symptoms of hyperglycaemia and an HbA1c > 9.0% [6]. A position statement by the American Diabetes Association (ADA) and European Association for the Study of Diabetes (EASD) also recommended the use of short‐term intensive insulin (STII) for the initial management of individuals with newly diagnosed T2DM if blood glucose is more than 300 mg/dL and/or HbA1c is more than 10%–12%, especially if the patient has symptoms of hyperglycaemia (polyuria, polydipsia, nocturia) or catabolic state (weight loss or ketosis) [7]. The use of STII in these patients has been associated with the early achievement of glycaemic control and improvement of pancreatic beta‐cell function [8]. It has been reported that up to 23% of patients have an HbA1c of more than 9% at the time of initial diagnosis [8]. Nevertheless, the treatment options should also consider important patient‐related factors such as the level of education, patient willingness to use insulin, ease of use, and affordability to ensure patient compliance. A significant number of patients are reluctant to use insulin. A study from Iran reported that up to 60.2% of the patients were reluctant to use insulin for DM management [9]. The most common reasons include financial constraints, fear of needles/pain and fear of dependency on insulin use [10].

Qatar has a population of 2.8 million, and the burden of diagnosed T2DM in Qatar is expected to increase from 7% to 14% from 2021 to 2050 [11]. A study by Abdullatef et al. reported the prevalence of newly diagnosed T2DM to be 21.1% in patients admitted with acute coronary syndrome (ACS) [12]. Our study aims to compare the glycaemic control in terms of hbA1c at 1‐year follow‐up between newly diagnosed DM patients with ACS who were discharged on insulin plus oral agents versus those discharged only on oral agents.

## 2 Methodology

### 2.1 Study Design

This study is an observational, single‐centre, retrospective study that included consecutive adults (age ≥ 18 years) with newly diagnosed T2DM with an hbA1c level ≥ 10% at the time of admission with ACS between 01/01/2020 and 30/08/2022. Patients were divided into two groups. Group 1 consisted of patients discharged only on oral anti‐diabetes drugs (OAD) and Group 2 included patients discharged on both insulin and OAD. Data were collected through electronic records (Cerner) for the included patients. Pregnant women, patients with pre‐existing T2DM, patients with T1DM, and those aged < 18 years were excluded.

### 2.2 Statistical Consideration and Data Analysis

Descriptive and summary statistics have been used to describe the study cohort's socio‐demographic parameters, with continuous variables presented as means (±standard deviation) or median (Interquartile range) as appropriate. In contrast, categorical variables have been presented as numbers (percentages). Pairwise comparisons between continuous and categorical study variables were done using a t‐test, Mann–Whitney test, and chi‐square. We used STATA 18 for data analysis. Baseline characteristics have been described for all the patients included in the study. In the analysis of outcomes at follow‐up, only patients who had follow‐up at 1‐year were included.

### 2.3 Ethical Declaration

The Medical Research Centre (MRC) at Hamad Medical Corporation, Qatar approved the conduct of this retrospective study (ID MRC‐01‐22‐605). The study has been performed according to the methods and principles of the Declaration of Helsinki. This study is not under consideration for publication in any other publication. Part of the results of this study have been presented as an abstract poster in the Eight Qatar Diabetes, Endocrine and Metabolic Conference 2024, and have been published in the abstract booklet [13].

### 2.4 Consent

Since this is a retrospective study, the requirement for informed consent was waived by the Medical Research Centre (MRC) of Hamad Medical Corporation, Qatar.

## 3 Results

A total of 149 patients were eligible for inclusion, of which the majority were males (97.3%) and South Asian (87.3%). The mean age was 47 ± 8.3 years. 43.6% patients (*N* = 65) had HTN, 14.8% (*N* = 22) were smokers and 45% (*N* = 67) were alcohol users. The baseline hbA1c at diagnosis was 11.2 (10.5–12.3) %, and the baseline BMI was 25.6 (23.3–27.9) kg/m^2^. Coronary angiogram showed single vessel involvement in the majority (*N* = 71, 48.6%) followed by 3 vessels (*N* = 36, 24.7%) and 2 vessel disease (*N* = 34, 23.3%). CABG was done in 15 (10.1%) patients. 38 (25.5%) were discharged on insulin + OADs, whereas 111 (75.5%) were discharged only on OADs. The majority were discharged on at least 3 non‐insulin agents (*N* = 109; 73.2%), of which metformin was the most common agent (*N* = 144, 96.6%), followed by DPP‐IV inhibitors (*N* = 129, 86.6%) and SGLT2 inhibitors (*N* = 122, 81.9%). 62 (41.6%) patients completed 1‐year follow‐up, whereas 87 (58.4%) did not follow‐up. There were no differences in terms of age (Mean +/− SD 46.9 ± 7.3 Vs 47.1 ± 9, *p* = 0.8), ethnicity (*p* = 0.3), comorbid conditions (*p* = 0.7), baseline BMI (Median (IQR) 26.2 (24.1–28.4) vs. 25.1 (23.1–27.5); *p* = 0.1), baseline hBA1c (Median (IQR) 11.5 (10.4–12.7) vs. 11.1 (10.5–12.2); *p* = 0.2) and type of discharge medications between the patients who completed 1‐year follow‐up compared to those who did not (*p* > 0.05).

Table 1 compares the outcomes of patients discharged only on OAD medications (Group 1, *N* = 48; 77.4%) to those discharged on OAD plus insulin (Group 2, *N* = 14; 22.6%). Patients only on OAD had a lower baseline hbA1c (Median IQR) 11 (10.25–12.15) vs. 13.5 (12.1–14.4)%, (*p* ≤ 0.001) and higher baseline BMI (Median (IQR) 27.3 (24.2–29.4) vs. 24.8 (22.8–26.2) kg/m^2^, *p* = 0.04) than those on OAD plus insulin. There were no differences in age (*p* = 0.3), ethnicity (*p* = 1), gender (*p* = 0.17), baseline TC (*p* = 0.7), TG (*p* = 0.9), HDL (*p* = 0.4), LDL (*p* = 0.2), number of vessels involved in CAG (0.08) and CABG (0.6) requirements between the 2 groups. There were no statistically significant differences in number of OAD prescribed between the 2 groups (*p* = 0.69). Regarding outcomes, there was no statistically significant difference in change in hbA1c from baseline between the two groups (Mean (SD) 4% ± 1.5% vs. 5.1% ± 2.4%, *p* = 0.07). Patients only on OAD had a lower TC (Median (IQR) 3.3 (2.6–4.4) vs. 4.6 (3.6–5.4) mmol/L, *p* = 0.02) and LDL (Median (IQR) 1.6 (1.2–2.7) vs. 3 (2–2.3) mmol/L, *p* = 0.02) at 12‐month than those on insulin plus OAD. None of the patients had any hyperglycaemic emergency, and there were no differences in recurrent admissions due to cardiac indications (*p* = 0.5).

**TABLE 1:** Outcomes of patients who completed 1‐year follow‐up (*N* = 62).

Variable	Units	All patients (*N* = 62)	Group 1 (only oral agents) (*N* = 48)	Group 2 (oral agents + Insulin) (*N* = 14)	*p*
Age, (Mean ± SD)	Years	46.9 ± 7.3	46.4 ± 7.3	48.6 ± 2	0.3
Gender	*N* (%)				
Male		58 (93.6)	46 (95.8)	12 (85.7)	0.17
Female		4 (6.4)	2 (4.2)	2 (14.3)	
Ethnicity	*N* (%)				
South Asian		52 (83.9)	40 (83.3)	12 (85.7)	1
Arab		8 (12.9)	6 (12.5)	2 (14.3)	
Others		2 (3.2)	2 (4.2)	0	
Alcohol use	*N* (%)	6 (9.7)	5 (10.4)	1 (7.1)	0.6
Smoking		27 (43.5)	22 (45.8)	5 (35.7)	0.5
Baseline BMI, Median (IQR)	kg/m^2^	27.3 (24.2–29.4)	27.3 (24.2–29.4)	24.8 (22.8–26.2)	0.04
Change in BMI (Mean ± SD)	kg/m^2^	−0.04 ± 3.8	−0.04 ± 3.7	−0.3 ± 4.4	0.8
Baseline HbA1c, Median (IQR)	%	11.5 (10.4–12.7)	11 (10.25–12.15)	13.5 (12.1–14.4)	< 0.001
Change in HbA1c (Mean ± SD)	%	4.4 ± 1.8	4 ± 1.5	5.1 ± 2.4	0.07
Number of vessels involved	*N* (%)				
Single vessel disease		30 (49.2)	21 (44.7)	9 (64.3)	0.08
Two vessel disease		19 (31.1)	18 (38.3)	1 (7.1)	
Three vessel disease		11 (18)	7 (14.9)	4 (28.6)	
CABG done	*N* (%)	4 (6.5)	3 (6.2)	1 (7.1)	0.6
Baseline lipid profile					
TC, (Mean ± SD)	mmol/L	5.2 ± 1.3	5.15 ± 1.3	5.3 ± 1.6	0.7
TG, Median (IQR)	mmol/L	2.25 (1.4–3.2)	2.2 (1.4–3.1)	2.7 (1.1–3.3)	0.9
HDL, Median (IQR)	mmol/L	0.8 (0.7–1)	0.8 (0.7–1)	0.9 (0.8–1.2)	0.4
LDL, (Mean ± SD)	mmol/L	3.15 ± 1.2	3.04 ± 1.2	3.5 ± 1.2	0.2
Oral/non‐insulin anti‐diabetes medications on discharge	*N* (%)				
Metformin		60 (96.8)	48 (100)	12 (85.7)	< 0.001
DDP4 inhibitors		53 (85.5)	41 (85.4)	12 (85.7)	0.7
SGLT2 inhibitors		52 (83.9)	40 (83.3)	12 (85.7)	0.8
Sulfonylureas		3 (4.8)	3 (6.2)	0	0.4
Thiazolidinediones		1 (1.6)	1 (2.1)	0	0.7
GLP‐1 agonists		1 (1.6)	1 (2.1)	0	0.7
Lipid Profile at 12‐month follow‐up					
TC, Median (IQR)	mmol/L	3.6 (2.74–4.6)	3.3 (2.6–4.4)	4.6 (3.6–5.4)	0.02
TG, Median (IQR)	mmol/L	1.4 (1.1–2.1)	1.4 (1.2–2.1)	1.4 (0.8–2.8)	0.8
HDL, Median (IQR)	mmol/L	0.9 (0.8–1.10)	1 (0.8–1.1)	0.9 (0.8–1)	0.7
LDL, Median (IQR)	mmol/L	1.75 (1.2–2.9)	1.6 (1.2–2.7)	3 (2–3.2)	0.01
Number of non‐insulin agents	*N* (%)				
1		4 (6.4)	3 (6.25)	1 (7.14)	0.69
2		11 (17.7)	7 (14.6)	4 (28.6)	
3		45 (72.6)	36 (75)	9 (64.3)	
4		1 (1.6)	1 (2.1)	0	
5		1 (1.6)	1 (2.1)	0	
DKA/HHS	*N* (%)	0	0	0	NA
Recurrent admissions due to cardiac reasons	*N* (%)	8 (12.9)	6 (12.5)	2 (14.3)	0.5

Table 2 compares patients who did not achieve target hbA1c < 7.5% (*N* = 20, 32.3%) to those who achieved the target (*N* = 42, 67.7%) at 12‐month follow‐up. Those achieving the target were younger (Mean ± SD of 45 ± 6.3 vs. 50.8 ± 7.8 years, *p* = 0.002). Patients not achieving the target had a higher baseline hbA1c (12.5 [11.3–14.1] vs. 11.05 [10.4–12.2]; *p* = 0.004) and included a greater proportion of patients discharged on a regimen consisting of insulin in discharge medications (40% vs. 14.3%, *p* 0.02).

**TABLE 2:** Comparison of patients who achieved target hbA1c at 1‐year follow‐up compared to those who did not achieve the target (*N* = 62).

Variable	Units	Target hbA1c not achieved (20, 32.3%)	Target hbA1c achieved (42, 67.7%)	*p*
Age, (Mean ± SD)	Years	50.8 ± 7.8	45 ± 6.3	0.002
Gender	*N* (%)			0.6
Male		18 (90)	40 (95.2)	
Female		2 (10)	2 (4.8)	
Ethnicity	*N* (%)			0.2
South Asian		16 (80)	36 (85.7)	
Arab		2 (10)	6 (14.3)	
Others		2 (10)	0	
Baseline BMI, Median (IQR)	kg/m^2^	24.9 (22.8–27.5)	26.5 (24.2–29.4)	0.06
Change in BMI, (Mean ± SD)	kg/m^2^	0.06 ± 3.2	−0.09 ± 4.1	0.8
Baseline HbA1c, Median (IQR)	%	12.5 (11.3–14.1)	11.05 (10.4–12.1)	0.004
Baseline lipid profile				
TC, (Mean ± SD)	mmol/L	5.3 ± 1.4	5.1 ± 1.3	0.6
TG, Median (IQR)	mmol/L	2.45 (1.4–4)	2.25 (1.4–3.1)	0.9
HDL, Median (IQR)	mmol/L	0.9 (0.8–1.2)	0.8 (0.7–1)	0.08
LDL, (Mean ± SD)	mmol/L	3.4 ± 1.1	3.03 ± 1.1	0.2
Insulin in discharge regimen	*N* (%)	8 (40)	6 (14.3)	0.02

## 4 Discussion

In this retrospective study including newly diagnosed T2DM patients with hbA1c > 10% at diagnosis on admission with ACS, 77.4% were discharged only on OAD (Group 1), while 22.6% were discharged on a regimen consisting of OAD and insulin (Group 2). There were no statistically significant differences in the change in hbA1c compared to baseline at the 1‐year follow‐up between the two groups. Patients discharged only on OAD had a lower TC and LDL at 1‐year follow‐up. Those achieving the target hbA1c were younger and were mainly discharged on a regimen consisting only of OAD.

As mentioned earlier, up to 29% of patients with acute MI might be newly diagnosed with DM at the time of admission [4]. Studies have shown that compared to patients without DM, patients with DM have a higher volume of atheroma, plaque burden and impairment in compensatory remodelling of arteries after an acute ASCVD event [14]. A linear correlation exists between higher hbA1c levels and ASCVD‐related mortality [15]. Patients with DM have a higher in‐hospital mortality and post‐infarction complications, including heart failure, angina and arrhythmias, compared to those without DM [16]. Hence, early control of glycemia is of paramount importance in patients after an acute coronary event and can lead to a reduction in mortality at 1‐year follow‐up [17]. This is of particular importance in patients who have very high hbA1c at the initial diagnosis, as uncontrolled diabetes is associated with an increased risk of ASCVD recurrence as well [18]. On the other hand, early intensive glycaemic control after DM diagnosis is known to have a legacy effect lasting for years after treatment and minimising the lifetime risk of DM‐related complications [19]. The choice of anti‐diabetes medications in these situations is of critical value. As explained earlier, guidelines recommend earlier initiation of subcutaneous insulin in outpatient regimens of patients with severely uncontrolled DM at diagnosis [7]. These recommendations are particularly applicable to patients who had a recent MI to achieve early glycaemic control post‐ASCVD. However, several patient‐related factors must be considered when choosing an anti‐DM regimen. Non‐compliance with insulin is a significant issue that clinicians encounter during the management of patients with DM, and any benefit of early insulin initiation with the aim of achieving early glycaemic control post‐MI can be offset by non‐compliance to insulin doses. Studies on insulin compliance have yielded variable results, with adherence rates between 52% and 70% depending on the cohort studied [20, 21]. Moreover, a significant proportion of insulin‐naïve patients are reluctant to initiate insulin. Several factors contribute to this, including fear of needles, inconvenience, negative impact on work, cost and affordability, and health beliefs, amongst many others [22, 23]. Moreover, there is a reluctance amongst patients to initiate insulin, with studies reporting insulin refusal rates between 35% and 74% [24, 25, 26]. In our cohort as well, only 25.5% of patients accepted insulin (in addition to OAD) as a discharge medication, leaving a significant concern for the probability of lack of early achievement of glycaemic control in such high‐risk patients. Hence, alternate safe and effective strategies must be searched to manage such cases.

Whether initiation of an alternative regimen consisting of multiple oral anti‐DM agents is safe and as effective as a regimen consisting of subcutaneous insulin is unclear. In our study, patients treated with OADs had a similar reduction in hbA1c from baseline compared to those with OAD plus insulin, providing evidence that an anti‐DM regimen consisting only of OAD can be a safe alternative in patients who are reluctant to use insulin. Our results are consistent with results from some other studies. In a retrospective study comparing OADs to early insulin initiation post‐DM diagnosis consisting of 94 patients in each group, Lee et al. found that oral OADs alone were non‐inferior to early insulin initiation. Moreover, in patients with higher baseline hbA1c (10.75%–10.92%), a greater hbA1c reduction and higher rates of well‐controlled glycaemic levels were noted in those taking OADs compared to the early insulin group [27]. In another study by Vaughan et al., an equal reduction in hbA1c at 12 months compared to baseline was noted in both oral agents and insulin groups amongst individuals with newly diagnosed DM with hbA1c > 11% [28]. All these results highlight the utility of a regimen consisting of multiple oral anti‐DM medications in patients with high hbA1c at diagnosis and ACS, in whom the early achievement of glycaemic control is of great value.

One main reason for recommending the initiation of an insulin‐based anti‐DM regimen in newly diagnosed DM patients with severe hyperglycaemia (glucose > 300 mg/dL or hbA1c > 10%–12%) is the understanding that such patients have insulinopenia and a state of glucose toxicity [29]. This also makes them prone to an increased risk of DKA development, as absolute or relative insulin deficiency is a cornerstone pathophysiological mechanism in DKA development. Initiation of insulin for a short term early after diagnosis in such situations improves glycaemic control and improves insulin secretion with long‐lasting effects on diabetes control [29, 30]. In our study, none of the patients discharged only on OADs had an episode of DKA during the 1‐year follow‐up period, again pointing to the safety profile of multiple oral agents as a safe alternative strategy for such patients. It is noteworthy that 83% of patients in the oral agents‐only group had SGLT2 inhibitors in the discharge prescription, and despite a theoretically increased risk of DKA in very uncontrolled newly diagnosed insulinopenic patients on SGLT2 inhibitors, no DKA occurred.

To our knowledge, this is the first study in the region comparing the use of OADs only to insulin + OADs in patients with acute ACS and who have very high hbA1c at DM diagnosis. With a high prevalence of newly diagnosed DM patients at the time of an ACS event and a high rate of insulin refusal, especially in treatment‐naïve patients, our results provide evidence for a safe and effective alternative treatment strategy for DM management based on data from real‐life experience. However, there are several limitations as well. There was a significant loss to follow‐up at 1‐year interval which led to a substantial reduction in sample size to evaluate the glycaemic outcomes at the 1‐year follow‐up. The nature of the patients who are non‐compliant with insulins or refuse insulin for other factors also share the behaviour of not coming for follow‐ups, and hence, any such study, if done retrospectively, will have a similar loss to follow‐up. However, as can be seen in Table 3, there were no differences in terms of ethnicity, baseline hbA1c, BMI, number of coronary vessels involved in ACS, treatment with CABG, types and number of oral agents, and number of patients discharged on insulin between patients who completed 1‐year follow‐up compared to those who were lost to follow‐up and hence, the results of the study can be interpreted without the significant danger of being affected due to loss to follow‐dataup. Another limitation is the lack of comparison of socioeconomic characteristics and the quality‐of‐life measures between the two groups of treatment regimens that could have further provided valuable information relevant to patient management. In terms of changes in hbA1c between the two groups, although the results did not show any statistically significant difference, the absolute difference in changes in hbA1c was 1.1% which may be clinically important. Hence, larger randomised controlled trials are needed to further assess this. Moreover, although the results showed that the proportion of patients achieving target hbA1c was higher if insulin was not part of the regimen, the baseline hbA1c was higher in patients who were discharged on insulin + OAD, which would have contributed to difficulty in achieving the target hbA1c. Another limitation of the study is the predominance of males in the study cohort. This is due to the fact that the majority of its population consists of expatriate workers, thus affecting the gender distribution to be predominantly males (74.1%) [31].

**TABLE 3:** Comparison of baseline characteristics of patients who completed 1‐year follow‐up compared to those who were lost to follow‐up.

Variable	Units	Total cohort (*N* = 149)	Follow‐up complete (*N* = 62, 41.6%)	Lost to follow‐up (*N* = 87, 58.4%)	*p*
Age, (Mean ± SD)	Years	47 ± 8.3	46.9 ± 7.3	47.1 ± 9	0.8
Gender	*N* (%)				
Male		145 (97.3)	58 (93.6)	87 (100)	0.028
Female		4 (2.7)	4 (6.4)	0	
Ethnicity	*N* (%)				
South Asian		130 (87.3)	52 (83.9)	78 (89.7)	0.3
Arab		13 (8.7)	8 (12.9)	5 (5.7)	
Others		6 (4)	2 (3.2)	4 (4.6)	
HTN	*N* (%)	65 (43.6)	26 (41.9)	39 (44.8)	0.7
Alcohol use	*N* (%)	22 (14.8)	6 (9.7)	16 (18.4)	0.1
Smoking		67 (45)	27 (43.6)	40 (46)	0.7
Baseline BMI, Median (IQR)	kg/m^2^	25.6 (23.3–27.9)	26.2 (24.1–28.4)	25.1 (23.1–27.5)	0.1
Baseline HbA1c, Median (IQR)	%	11.2 (10.5–12.3)	11.5 (10.4–12.7)	11.1 (10.5–12.2)	0.2
Number of vessels involved	*N* (%)				
Single vessel disease		71 (48.6)	30 (49.2)	41 (48.2)	0.1
Two vessel disease		34 (23.3)	19 (31.1)	15 (17.6)	
Three vessel disease		36 (24.7)	11 (18)	25 (29.4)	
Not mentioned		2 (1.4)	1 (1.6)	1 (1.2)	
CAG not done		3 (2)	0	3 (3.5)	
CABG done	*N* (%)	15 (10.1)	4 (6.4)	11 (12.6)	0.3
Oral/non‐insulin anti‐diabetes medications on discharge	*N* (%)				
Metformin		144 (96.6)	60 (96.8)	84 (96.6)	0.9
DDP4 inhibitors		129 (86.6)	53 (85.5)	76 (87.4)	0.7
SGLT2 inhibitors		122 (81.9)	52 (83.9)	70 (80.5)	0.6
Sulfonylureas		9 (6)	3 (4.8)	6 (6.9)	0.7
Thiazolidinediones		1 (0.7)	1 (1.6)	0	0.4
GLP‐1 agonists		1 (0.6)	1 (1.6)	0	0.4
Number of non‐insulin agents	*N* (%)				
1		8 (5.4)	4 (6.4)	4 (4.6)	0.8
2		29 (19.5)	11 (17.7)	18 (20.7)	
3		109 (73.2)	45 (72.6)	64 (73.6)	
4		2 (1.3)	1 (1.6)	1 (1.1)	
5		1 (0.7)	1 (1.6)	0	
Insulin on discharge	*N* (%)	38 (25.5)	14 (22.5)	24 (27.6)	0.49

## 5 Conclusion

In newly diagnosed DM type 2 patients with ACS who refuse to take insulin, an anti‐DM regimen consisting of multiple oral agents is a safe and effective alternative and can lead to a comparable reduction in hbA1c at 1‐year follow‐up without a concomitant increase in the risk of acute hyperglycaemic events and recurrent admission due to cardiac reasons. Larger prospective studies are required to further validate results.

## Author Contributions

A.A.K.: conceptualisation, data curation, formal analysis, investigation, methodology, project administration, validation, visualisation, writing – original draft, writing – review and editing. F.A.: formal analysis, investigation, visualisation, writing – original draft. A.A., A.J.Q., A.S., K.A.A., Y.A.: data curation, writing – original draft. A.J.: Literature review, data curation, manuscript writing. M.B.: formal analysis, methodology, validation. H.S.: conceptualisation, supervision, writing – review and editing.

## Conflicts of Interest

The authors declare no conflicts of interest.

## Data Availability

The data sets generated and analysed during the current study are available from the corresponding author upon reasonable request.
